# Using Gesture and Speech to Control Surgical Lighting Systems: Mixed Methods Study

**DOI:** 10.2196/70628

**Published:** 2025-05-12

**Authors:** Nima Zargham, Anke V Reinschluessel, Andre Mühlenbrock, Thomas Muender, Timur Cetin, Verena Nicole Uslar, Dirk Weyhe, Rainer Malaka, Tanja Döring

**Affiliations:** 1 Faculty of Mathematics and Computer Science University of Bremen Bremen Germany; 2 Department of Computer and Information Science University of Konstanz Konstanz Germany; 3 Department of Human Medicine University of Oldenburg Oldenburg Germany; 4 Pius Hospital Oldenburg Oldenburg Germany

**Keywords:** speech interaction, gesture recognition, operating theater, surgical lighting systems, smart lighting, artificial intelligence

## Abstract

**Background:**

Surgical lighting systems (SLSs) provide optimal lighting conditions for operating room personnel. Current systems are mainly adjusted by hand; surgeons either accommodate the light themselves or communicate their requirements to an assistant to ensure optimal surgical conditions. This poses challenges to maintaining sterility, proper accessibility, and illumination and can lead to potential collision problems. Furthermore, the personnel operating the light may not have deep medical knowledge or equipment expertise.

**Objective:**

This paper introduces a touch-free interaction concept for controlling an SLS using speech and gestures.

**Methods:**

We used an iterative, user-centered design approach with participatory design sessions. This process involved conducting a literature review, several observations of actual surgical sites, and engaging stakeholders through interviews and focus groups. In addition, we carried out 2 user studies: one in a virtual reality setup and another in a living laboratory environment.

**Results:**

Our findings indicate that our interaction concept is a viable alternative for controlling an SLS. Despite some technical limitations, surgical experts found the system intuitive and useful, recognizing the significant potential for touch-free lighting adjustments in the operating room. The combination of speech and gesture modalities was seen as helpful and even necessary, with some interactions better suited to one modality over the other. Offering both modalities for each interaction provided greater flexibility.

**Conclusions:**

Our findings suggest that our proposed touch-free interaction concept can enhance surgical conditions and has the potential to replace traditional adjustment.

## Introduction

### Background

The illumination of the operating room (OR) is critical to ensuring optimal surgical conditions. Surgical lighting systems (SLSs) are designed to provide optimal lighting for the surgical staff in the OR. They provide settings for adjusting the intensity of the light, its color temperature, and the size of the illuminated field, which are mainly controlled with control panels situated on the lamp’s body. Such adjustments to the SLS are necessary for various reasons, such as differentiating between only marginally different-looking tissues [1]. Making modifications to the lighting conditions requires the surgeon or an assistant to operate the control panel with their hands, which is often located in an ergonomically unfavorable position, such as above the surgeon’s head [2].

Generally, an OR is required to have two sets of distinctive lighting types: (1) general room lights (ambient lights) and (2) focused and precise procedural surgical lights to illuminate areas of incision (surgical lights) [3]. Optimal surgical illumination combines sufficient ambient light with the ability to apply focused light at specific positions and angles throughout different stages of an operation [4]. Current SLSs are primarily controlled and adjusted by hand, and surgeons are eager to operate the SLSs themselves while keeping sterility. It is essential to look for an ergonomic solution for the SLS adjustments to maintain the surgical staff’s focus on their primary task and keep distractions to a minimum. Hence, controlling the SLS should be intuitive and straightforward to keep the mental load to a minimum and increase interaction safety [1]. Moreover, during an operation, the surgical staff must reposition the SLS several times to ensure optimal surgical illumination. Research shows that every 7.5 minutes, a change in the lighting position takes place [2]. Such position changes are also commonly performed by hand. However, performing such adjustments by hand can lead to collisions and other mechanical problems. In addition, this raises concerns regarding sterility and increases potential safety risks [5]. Even though it is required to sterilize all equipment the surgical staff comes in contact with during an operation, this still might not be done sufficiently due to the lack of information and attention of the OR personnel, use of the wrong sterilization product, or insufficient and ineffective work of sterilization [5]. During an operation, surgeons often need to interact with the OR equipment, such as radiological images and lights [6]. Due to sterility concerns, surgeons may be unable to interact with such equipment and depend on their assistants. Communicating this need with the personnel might be complex and lead to further errors if the assistant and the surgeon cannot communicate properly. Consequently, there is a possibility of interruptions in the workflow.

In this work, we propose the use of natural user interfaces (NUIs) [7,8] to provide surgeons with an intuitive interface for adjusting the SLS. NUIs have the potential to enable clinicians to have freehand control of the OR equipment while maintaining sterility. Data from several studies highlight the potential and advantages of using NUIs in ORs [1,6,9-13]. This paper presents an interaction concept that combines voice and gesture to control the SLS. We used a touch-free approach to protect sterility in the OR and ultimately increase patient safety. To develop this interaction concept, we used an iterative user-centered design approach with participatory design sessions. First, we conducted a literature review and had several observations of actual surgical sites to develop the initial list of interactions. Using this list, through co-design, we discussed individual interactions for both modalities of speech and hand gestures with 7 experts using interviews and developed an initial interaction concept. Subsequently, we conducted 2 focus groups to discuss the concept with the OR personnel. The revised interaction concept based on the focus groups’ feedback was then used in a virtual reality (VR) prototype and evaluated by 6 experts. Considering the results of the VR evaluation, we developed a prototype in a living laboratory and conducted a laboratory study with 12 surgeons and surgical assistants. Our findings suggest that our interaction concept is a valid alternative for controlling the SLS. Even though certain technical limitations were experienced, surgical experts found the system highly useful and intuitive and saw great potential for touch-free approaches to lighting adjustments in the OR. This paper contributes the following: (1) the design and implementation of a touch-free interaction system for controlling the SLS informed by domain experts and (2) 2 user studies providing an exploratory evaluation of how this system can support surgical personnel. The implications of our work can support researchers and medical companies when adapting touch-free approaches for adjusting SLS. It further gives an understanding of the potential and challenges of such approaches. The broader insights of this research can also be valuable for the general use of NUIs in the OR to enhance surgical conditions and increase patient safety.

All figures used in the manuscript can be viewed in Multimedia Appendix 1.

### Related Work

Many OR personnel believe that lighting is a major inconvenience [14]. Previous literature identified the following types of problems with SLS: *mechanical*, *collision*, *accessibility*, and *illumination* problems [2,15,16]. *Mechanical* problems include the requirement of excessive force to move the SLS, which can make a 1-hand light adjustment difficult and occasionally cause the SLS to get jammed. In such cases, surgeons usually ask for the help of other OR personnel. This can delay the completion of the adjustment and could lead to possible errors because the personnel responsible might not have the necessary medical knowledge or familiarity with the equipment [2]. *Collision* problems refer to the times when the SLS collides with other OR equipment or against the heads of the personnel [2]. *Accessibility* problems occur when surgeons have to stand up or move to perform an adjustment because the SLS is out of reach [2]. *Illumination* problems refer to the issues that the surgeons or the surgical staff experience regarding the SLS lighting conditions, such as wound illumination, refocusing the light beam, and increasing illumination levels [15].

In the following sections, we provide an overview of the previous literature on smart surgical light control and touch-free techniques for an OR, including voice and gesture interaction in the OR.

### Smart Light Control in the OR

Appropriate lighting during surgery is critical for patient safety [17]. Researchers have looked into various methods to enhance the lighting conditions in the OR and the interaction with the SLS. Teuber et al [18] presented a method for autonomous positioning of surgical lamps during open surgeries using a depth camera and robotic arms. The authors tested their algorithm in a VR simulation and found that their method was robust and could ensure close-to-optimal lighting conditions in real-world surgeries. Burger [15] developed a technique for autonomous, real-time adjustment of an SLS by identifying the wound based on heat sources to achieve complete automation of an SLS in the OR. The findings suggested that the combination of optical and thermal cameras with stereo image-processing techniques could be used to identify and track a heat source. Moreover, predefined scene lighting has been commonly examined and used for medical environments, such as angiography rooms [3]. This is accomplished by defining specific lighting features, such as the light position, intensity, angle, and color. Mühlenbrock et al [19,20] introduced a novel lighting system consisting of numerous small lighting modules permanently mounted on the ceiling. This system actively avoids casting shadows using depth sensors. Through simulations and a subsequently constructed prototype, which was evaluated with surgeons, it was demonstrated that in open abdominal surgeries, this system can illuminate a surgical wound more effectively than conventional surgical lights and was deemed more suitable for use in the operating theater by surgeons. In addition, point cloud recordings were used to investigate how the positioning and optimization of these light modules affect the achievable brightness at the surgical site [21]. Other studies have shown the potential of freehand control for operating the OR lights. Hartmann and Schlaefer [9] used an RGBD (red, green, and blue, plus depth) camera for gesture tracking to study gesture-based control of OR lights. Their results showed that gesture control could be quickly learned and effectively used to operate OR lights. Similarly, to offer the surgeons a sterile and ergonomically comfortable operation, Dietz et al [1] developed a freehand gesture control using a Kinect (Microsoft Corporation) system for the SLS. Participants reported high usability scores for the system and rated it ergonomically more comfortable than the conventional control panels and less likely to distract them from their tasks. In this work, we build on the lighting modules introduced by Mühlenbrock et al [20] in our living laboratory study, implementing a freehand control method to adjust the lighting conditions in the OR.

### Touch-Free Techniques in the OR

An extensive body of literature has previously looked into touch-free methods for controlling OR equipment. As input modalities, gaze [22-24], gestures [12,22,25-33], or voice [11,34-36], for example, were researched. Hatscher et al [22] used gaze tracking and foot gestures on an interactive floor as input modalities for hands-free interaction to interact with medical image data. Their results show that selection is accomplished faster via gaze than with a foot-only approach, but gaze and foot easily interfere when used simultaneously. To minimize the dependency on OR personnel, Zaman et al [35] investigated voice- and foot-based interactions for the surgeon to interact with 2D images in VR. Their findings suggest that both modalities have acceptable usability. While the voice command system was perceived as more comfortable, foot-based interaction was more efficient.

The use of voice technology in health care is growing. Research has shown that voice-based commands could lower the cognitive and physical cost of accessing specific features or performing certain actions [37]. Feedback from experts revealed that surgeons have previously requested more voice-controlled features in ORs, including voice commands for adjusting lamp positions [14]. Often, in critical situations during an operation, the surgeon and the assistants must use both hands to ascertain the patient’s health [38]. In this context, the advantage of voice interaction is that the surgeons can concentrate on the operation while simultaneously controlling other components using their voice [38,39]. The most crucial point is that the surgeon’s hands are always unencumbered. Previous research has looked into the use of voice interaction in an OR [36,40-43]. El-Shallaly et al [40] evaluated a voice interface regarding the use of time and surgical staff during laparoscopic cholecystectomy. Their results showed that the voice interface significantly optimized the operating time and the use of surgical staff. In a comparison study, Alapetite et al [39] compared a voice interaction system with the traditional touchscreen and keyboard interface to update anesthesia records during crisis situations. Their results showed an accurate speech-based registration performance even during emergencies and time-critical scenarios. Punt et al [44] compared the efficiency, reliability, and user satisfaction of controlling an endoscope’s zoom and light intensity using voice control, a touch panel, or manual control by an assistant. Most participants experienced voice control as the quickest interface, although in reality, it was slower than the other 2 conditions. Recent advances in natural language processing and artificial intelligence have extensively enhanced the efficacy of such systems, addressing some of the most prominent concerns with these systems, such as speech recognition. These advances provide new opportunities to explore the use of voice in various domains, including health care [45]. The emergence of large language models and generative artificial intelligence technologies such as ChatGPT (OpenAI) has ushered in notable improvements in this regard [46]. Another commonly proposed modality for hand-free interaction is gesture control. Gesture-based systems have been considered a novel application to adjust medical equipment [32,33]. Previous research has looked into navigating magnetic resonance imaging and computer tomography images using arm gestures during surgery [6,13,31]. Nestorov et al [30] designed a touch-free medical image control system using Leap Motion (Leap Motion, Inc) and Kinect. Their results showed an average acceptability rate for both systems. Nonetheless, surgeons and radiologists found Kinect to have better utility and be more beneficial for most clinicians. Overall, each of these modalities has demonstrated potential in ORs, improving the working conditions for surgeons. Researchers have also investigated the combination of different interaction modalities. Data from several studies suggest that incorporating voice and gesture inputs can be helpful in the OR [10,29,42]. To operate a system without the risk of equipment contamination, Ebert et al [10] developed a system prototype that allows for touch-free control of medical images using a depth camera and voice recognition software. The authors found that the touch-free system performs favorably and removes a potential vector for infection, protecting both patients and staff. Similarly, Hötker et al [42] demonstrated the feasibility and utility of using speech and gesture for reviewing images in interventional radiology to navigate through image data in a sterile environment. Their results showed high-command recognition rates and stability under different lighting conditions. In a study investigating the uses of voice control versus gestural control in the OR, Mentis et al [11] designed a system that allows for both modalities at the user’s choice.

Authors suggest that the benefits of each technology are circumstantial and recommend enabling functionalities to be achieved using both modalities to have a more flexible combination of their benefits as the context of the procedure requires [11]. Previous studies have highlighted that voice interaction is better fitted for trigger functions or functionality switches, whereas hand or body gestures are more suitable for continuous manipulation of parameters [11,38]. Although alternative methods for controlling the OR equipment have been largely investigated, to our knowledge, using speech and gesture to operate the SLS has yet to be evaluated in this context. Moreover, previous literature still lacks an understanding of the experts’ expectations and perceptions of such systems. Building on the previous work, in this paper, we engage with OR personnel to design a touch-free interaction concept for controlling the SLS in an iterative user-centered approach.

## Methods 1: Initial Concept

### Overview

In this work, we sought to develop and evaluate a touch-free interaction concept for controlling an SLS. For this, we used an iterative, user-centered approach to address the needs and requirements of the OR personnel. We could not directly translate existing touch-free practices to controlling an SLS, as the use case in the OR is unique due to its constraints (eg, patient safety or sterility aspects) and requires extra attention. Therefore, we chose a human-centered approach to best integrate and fulfill the needs of the OR personnel. The iterative process allowed us to enhance the concept by reevaluating it several times. This approach also helped us identify possible weaknesses and issues at the early stages. Furthermore, it enabled us to fill the gap between our intended design and how the experts interpret it [47,48]. We first sat in on an actual surgery to observe how surgical personnel interact with the SLS; on the basis of the observations and related work, we curated a list of required SLS interactions, which were discussed with experts and further refined using a focus group.

### Assessing OR Light Adjustments

To come up with our initial concept, we first reviewed the existing literature to explore previous work on contactless approaches for controlling surgical equipment [1,10,29,38,42]. A short summary of our review is mentioned in the Related Work section of this paper. Furthermore, to better understand how the OR personnel, especially the lead surgeon, interact with the current surgical lights, we attended an open liver surgery. The main goal was to identify what type of interactions occur with the lights, which personnel modify the SLS, how often it is adjusted, and how the adjustments are made throughout different phases of an operation. In total, 7 surgical personnel were involved in this surgery. The operation took around 81 minutes. For this surgery, 2 satellite lamps were used as focused lights to illuminate the wound. During this time, 21 light adjustments were made (6 major and 15 minor), that is, approximately 1 adjustment for every 4 minutes. Major light adjustments involve significant changes to overall illumination or focus to accommodate procedural needs, while minor adjustments are precise tweaks to optimize visibility without altering the general lighting setup. We observed that all the light adjustments were made using a single hand during the surgery. The reasons for light adjustments were mainly due to the lead surgeon or assistants changing poses, using other OR equipment, such as the ultrasound device, or changing the light’s focus. Only around 35% of adjustments took the shortest route to get to the next position. The lead surgeon only adjusted the SLS 20% of the time. Three other assistant surgeons made the remaining adjustments. We observed that light adjustments led to interruptions and interference with the personnel’s task at hand. For instance, the lead surgeon had to stop what they were doing to adjust the light and then continue with the work. Considering our literature research and observation of the surgery, we developed a list of possible interactions to perform with the SLS, which includes the *light switch*, *light beam position*, *size of the illuminated field*, *light intensity*, and *predefined lights*. On the basis of the recommendations from the previous research, we chose the 2 modalities of *speech* and *gesture* to use for our touchless interaction concept.

### Expert Interviews

After developing a list of possible interactions with the light, we conducted 7 interview sessions with the OR personnel to better match our concept to their needs and discuss our modalities and individual interactions, with the aim of designing an initial interaction concept.

#### Procedure

All interview sessions were held remotely via video calls while the experimenter recorded verbal statements and observations. The participants were asked to give informed consent and fill in the demographics questionnaire before the session. Participants were briefed about the interview procedure at the beginning of each session. We explained our concept and research goals, including our intention to use voice and hand gestures to control the SLS. We first asked about the experts’ impression of the concept when the interview started. After that, they provided feedback about each interaction and its positive or negative aspects. We then asked for their suggestions to enhance the current concept. For further qualitative analysis, we audio-recorded the sessions. The interview sessions took between 35 and 45 minutes. The interview recordings were summarized, then analyzed and coded based on domain summaries [49,50], where the themes are structured around a shared topic rather than shared meaning, with the goal of capturing the diversity of meaning in relation to a specific subject or area of focus [51]. The transcripts of the interviews were coded by a researcher using inductive coding [52,53], where a single quote could be assigned to multiple codes. In addition, we collected insightful and unique statements.

#### Participants

We recruited 7 senior physicians (1 female and 6 male) who were aged between 41 and 58 (mean 47, SD 6.16) years, all with at least 17 (mean 22, SD 5.12) years of medical experience and active roles in surgeries. Moreover, 4 were general and visceral surgery specialists, 1 was a neurosurgeon, 1 was a urological surgeon, and 1 was an ear-nose-throat specialist.

### Ethical Considerations

Ethics approval was received for every step of our evaluation from the University of Oldenburg ethics board. For each step, participants gave informed consent before participation. All data collected during the study were anonymized following the sessions to ensure participant confidentiality and privacy. No personally identifiable information was stored or retained. Participation in the study was entirely voluntary, and no compensation was provided.

## Results 1

### Overview

The experts generally liked the idea of using voice and gesture to control the SLS. They found it beneficial to have more control over the lights and avoid having additional personnel to make such changes. One expert said, “It is exhausting to ask an assistant to control the light. It takes extra effort” (P1). Another mentioned, “Everything you have to ask somebody else to do for you should have smart control” (P4). Furthermore, one expert pointed out that using such a concept can reduce unnecessary movement in the OR, for example, moving around for activities such as regulating or switching the lights. All participants agreed that multiple personnel should be able to adjust the SLS. This includes the anesthesiologist, the instrument nurse, and the assistant surgeons. One participant suggested that restricting access to certain personnel in special cases could be helpful. One participant suggested designing the interactions based on what is commonly used in ORs, such as similar sentences or phrases that are now used as an assistance request, “Easiest way is always to follow what you have done before” (P3). Two other experts recommended designing familiar gestures based on real-world interactions. For our experts, minor position changes were the most critical interactions often used during surgeries. Four participants mentioned that major position adjustments are less often performed in surgeries than fine adjustments, which matches our observations. Regarding the light intensity, 4 found it helpful to adjust the light intensity intraoperatively. On the other hand, 3 found it unnecessary to adjust the brightness of the SLS. In addition, 6 out of the 7 participants agreed that using predefined lights is beneficial. Participants found it helpful to record specific light characteristics to be able to reload them again afterward. One expert said, “It is an absolutely useful feature to save specific light positions and go back to it later” (P2). Another participant mentioned, “It would be beneficial if we could define certain positions based on the positions of the organs and just load them” (P3). One expert judged predefined lighting conditions to be an unnecessary feature. Furthermore, 3 participants suggested changing the light color to be helpful during the surgery. One surgeon highlighted that using the color green during laparoscopic surgery is quite beneficial, and it would be helpful to make this change with the new concept. Participants also pointed out possible concerns regarding each modality. Although all participants gave highly positive feedback on the concept, some were uncertain about the efficiency of the technology. Five participants were concerned about the noise level in the OR and whether voice input could be used in such an environment. Participants were worried about the recognition of the gestures and speech commands. Two mentioned that the unrecognized commands could frustrate the personnel and add more stress. Four participants were worried about misrecognition and false positives where the OR personnel give input, but the system does something otherwise. Moreover, participants raised concerns about having multiple hands near the wound, which might be problematic for gesture recognition. In addition, 6 out of the 7 participants suggested using a wake word for the voice input to minimize false recognition by the system. Four experts emphasized the importance of choosing a suitable wake word, suggesting using words or phrases not commonly used in the OR. A similar initiation step was also recommended for the gesture interaction to avoid false recognition. Moreover, 2 participants pointed out the importance of considering lighting scenarios that different surgical disciplines can use. Experts mentioned that designing smart lighting systems may not suit every type of surgery. For instance, the positions are typically predefined in spinal surgeries, and there is little variation. Moreover, in some instances, the surgeons use different lighting sources, such as headlights.

### Initial Concept

#### Overview

On the basis of the discussions and findings from the expert interviews, previous literature, and technical considerations, we developed an initial interaction concept (Table 1).

**Table 1:** Interactions in the initial concept with their respective modality and action to perform the interaction.

Interaction	Modality	Action
System activation	Speech	“Activate”
Light switch	Speech	“Surgical lights on”
Light beam position (minor)	Gesture	Move hand for positioning
Light beam position (major)	Speech	“Position three”
Light intensity	Speech	“Intensity 75 percent”
Size of the illuminated field	Gesture	Distance between the two hands
Predefined positions	Speech	“Standard mode”
Previous position	Speech	“Previous position”

#### System Activation

To interact with the system, the system first needs to be activated using a wake word. This activation is done via speech using the command “activate.” After using the wake word, there is a 5-second window to interact with the system. The system returns to standby mode when an action is performed, awaiting the next wake word. If no action happens, the system returns to standby mode after 5 seconds.

#### Light Switch

Switching the lights on and off is done using speech. Users can turn the SLS on by saying, “surgical lights on!” and, similarly, switch it off by saying, “surgical lights off!”

#### Light Position

The major position changes are done using speech interaction. The possibility is to either use numbers for each major position (eg, “position two”) or specific names (eg, “position stomach”). The surgical team can add custom positions with their desired names for that position. Fine (ie, minor) adjustments are performed with a combination of speech and gestures. After the wake word, users must use the command “edit position!” The system will then follow the personnel’s hand. After finding the right spot, the position can be set by saying “save position!” The position is saved afterward, and the hands are no longer tracked as the system returns to standby mode. The personnel can always command the system to return to the previous position by saying, “previous position.”

#### Light Intensity

The intensity of light is also adjusted by speech. Users can use percentages to modify the intensity, for example, “intensity 75 percent.”

#### Predefined Settings and Modes

Different predefined illumination modes can be set where specific lighting conditions are saved for multiuse purposes. For instance, in dark mode, the lights are off, and the personnel can better focus on the information from the monitors (eg, the ultrasound device). The modes are set using speech, where users can give commands such as “standard mode,” “dark mode,” or “laparoscopic mode” (which turns the light green).

#### Field of Illumination

To adjust the size of the illuminated field, we chose a combination of hand gestures and speech. After system activation, users must use the command “edit angle.” The system would then track the surgeon’s hands and calculate the distance between them. The more distant they are, the wider the angle, and vice versa. After finding the right size, the position can be set by saying “save.” The position is saved afterward, and the system returns to standby mode.

## Methods 2: Focus Groups

### Overview

**Figure 1:**
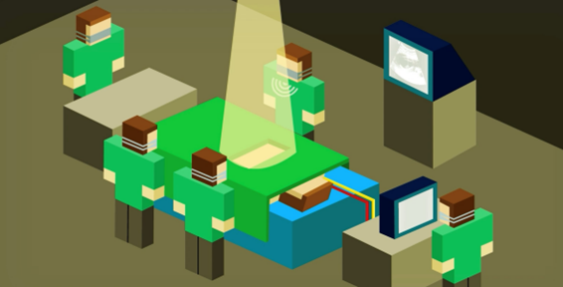
A frame from the concept animation that was used in the focus group sessions to help visualize the interactions for the experts.

We conducted 2 focus groups with the surgical personnel to evaluate and discuss our initial interaction concept. We used an approach similar to scenario-based design methods [54] and vignette experiments [55]. We developed animated videos demonstrating the interactions based on the concept (Figure 1). In these videos, we visualized how each interaction could be done in the OR using the previously defined and described input modalities. This allowed us to investigate the interaction and technologies without worrying about technological concerns. The main goal for the focus group sessions was to design the input format for each interaction, including individual voice commands and different hand gestures. Furthermore, we wanted to discuss the appropriate modality for individual interaction.

### Procedure

**Figure 2:**
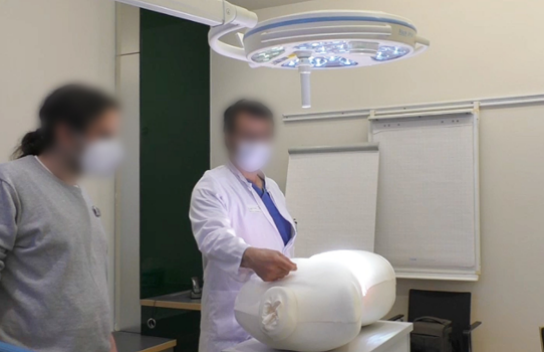
A surgeon acting out the interactions in the first focus group session.

We conducted 2 focus groups, inviting different target groups. Both sessions were conducted in a hybrid format. The first focus group (FG1) consisted of 6 surgeons (2 female surgeons and 4 male surgeons), all present in a hospital meeting room, with 2 researchers assisting them in person (Figure 2). Two other researchers joined in via video call. The second focus group (FG2) was conducted with 4 surgical assistants (one female surgical assistant and 3 male surgical assistants). Similar to the first session, all participants and 3 researchers were present in a hospital meeting room, and 1 researcher joined via video call. We grouped surgical assistants separately from surgeons to allow for a comfortable reflection without the potential influence of the hierarchical structure within surgical teams. Both sessions were video recorded for further analysis. Participants gave informed consent and filled in a demographics questionnaire at the beginning of each session, which was followed by a brief overview of the session’s procedure. Our interaction method was then presented along with the chosen modalities as well as the primary research goals. Next, individual interactions and possible input forms for each were discussed. The participants then acted on these suggestions in Wizard-of-Oz style, where they would make hand gestures or say the speech commands and the researchers would modify the lamp to simulate the interactions so that the participants could experience their suggested interactions better. Afterward, we demonstrated our animated concept video and collected the participants’ feedback. We discussed the animated videos after the initial discussion about the interactions to avoid biasing participants with our chosen interaction set and modalities. In the end, we left the floor open for further comments or suggestions from the experts. The first session took approximately 90 minutes (FG1), and the second took approximately 80 minutes (FG2). On top of the video recordings in each session, 2 researchers noted verbal statements and their own observations. The researchers reviewed, analyzed, and summarized the session recordings of both focus groups.

## Results 2

### Overview

The focus group results generally mirrored those obtained from interviewing experts. The focus groups led to further insights regarding participants’ concerns with the system and how such systems should be designed. Participants again pointed out that the interactions should be familiar to the personnel: “It would be nice to have a similar approach like the current way of moving the lamps” (P2 in FG1). All participants agreed that multiple personnel should be able to access the system. One participant mentioned, “When both surgeon’s hands are occupied, an assistant should be able to control the light” (P5 in FG2). In both focus groups, experts agreed that the interactions should be kept as simple and short as possible. Participants believed it would be valuable if the new interaction concept could help shorten the operating time.

### Appropriate Modality

Three participants agreed that speech would be more practical than gestures overall, as they believed gestures could be more distracting and lead to collisions. Participants agreed that speech would be more suitable for switching the lights on or off. All recommended that hand gestures should be used to perform fine adjustments. Regarding brightness and field of illumination, there were mixed feelings. Two participants believed speech would be more appropriate, while others suggested using gestures. The feedback about adjusting the size of the illuminated field varied among participants. Some believed this was best performed by speech using some sort of a scale, such as percentages, while others thought it would be more accurate using hand gestures. Two participants highlighted that the hand gestures should all be made using 1 hand. They believed that occupying both hands to adjust the SLS could delay the operation. The participants of both focus groups mainly agreed that position changes should be done with hand gestures, as they seem more precise. It was difficult for the participants to imagine speech commands that could accurately and quickly adjust the position of the light’s focus.

The general agreement in the sessions regarding the voice commands was that they needed to be as short as possible. Experts believed that more extended commands would be harder to remember. More importantly, brief commands would save time, a critical aspect of surgeries. Using phrases not commonly used in the OR was recommended to avoid false recognition. There was uncertainty about the language used for the speech commands. Three participants believed the commands should be given in the local language (German), as it is easier to learn, remember, and correctly pronounce them. Others suggested using a language different from the local one, such as English, because it is not commonly spoken in the OR. It was recommended that the system should be flexible and able to handle multiple phrases per action, meaning multiple commands should be able to perform the same interaction.

### Individual Interactions

We received multiple suggestions for a wake word, including “sun,” “sunlight,” “OP light,” and “light.” Among these, “light” was favored by most as it is short and easy to remember. Moreover, it was recommended that an activation step be used for hand gestures to avoid false positives. One participant suggested holding a gesture for a specific amount of time before giving the actual gesture. One participant recommended simulating pressing a light switch with hand gestures to switch the lights on and off. Another suggested opening and closing a fist to switch the lights. Participants mainly agreed that “lights on” or “lights off” are appropriate commands for speech regarding the light switch. For positioning the light beam, 2 participants tried to grab the beam of light and move it to a different position. Others in the group also found this form of interaction natural. Participants had difficulty imagining an appropriate speech command for positioning. One participant suggested using distance and a direction (eg, “20 centimeters left”), but others found this complex and time-consuming. Participants mainly agreed that having the possibility to go back to the previous position could be beneficial during operations. Regarding light intensity, one of the participants recommended using percentages. Another suggested using numbers from 1 to 10. For gesture interaction, a participant suggested moving a fist up and down to adjust the light intensity, similar to dragging a slider. Similar to the light intensity, numeric commands were recommended for speech input to adjust the illumination field. For gestures, 1 participant suggested using both hands in an L-shaped form (fully opened thumb and index finger, while other fingers are closed) and moving them closer or further away to make adjustments. The group also favored this, although it did not follow the one-hand-in-use recommendation. Predefined light settings were highly favored by the participants. All participants agreed that such a feature could be valuable for the OR and could help save time. One participant said, “We can use a specific setup and call it ‘scenario 1’, and then just say ‘scenario 1’ if we want to use this setting” (P3 in FG1).

### Feedback on Animated Videos

Participants commented on the initial interaction concept based on the animated video. They generally liked the interactions and found them intuitive. Experts found some speech commands long and highlighted that they must be quick and short. Participants found the wake word sufficient but suggested using their recommended phrases instead. They found it appropriate to switch the lights with speech. Similarly, participants believed that modifying the light’s position with hand gestures is intuitive and would be challenging with speech. Nevertheless, they found it helpful to use speech for predefined positions, including the previous position. It was mentioned that hand gestures used for the concept are easy to remember. Two participants recommended adjusting the system with both modalities to give the personnel more options and to allow them to choose the best based on circumstances.

### Concerns

Similar to the interviews, experts had concerns regarding the reliability of the technology. They were skeptical about the accuracy of recognition of both speech and gesture. One expert mentioned, “It is critical to lower the stress level in the OR. At the moment, with this technology, I feel a bit stressed.” (P1 in FG1). Participants were worried about the use of speech in the OR due to the high noise level. They repeatedly noted that surgeons may be unable to adjust the lamps as accurately as the current lights. Furthermore, 2 participants were concerned about the additional cognitive load on the surgical personnel caused by complicated interactions. They were worried that the surgical personnel might not remember the commands quickly enough in a stressful situation. Three participants suggested having a backup control for such situations. Regarding speech, 1 participant was skeptical about recognizing commands with different dialects.

### Adjustments to the Interaction List

**Figure 3:**
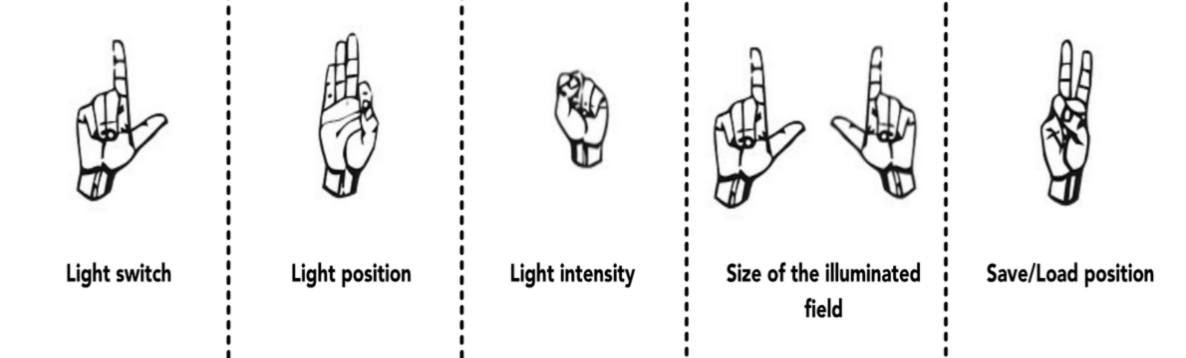
Interactions in the virtual reality prototype for gestures.

Considering the insights of the interviews and focus groups, we refined the interaction concept. Regarding speech commands, we shortened some of the commands and changed the wake word based on the expert recommendations to “Hey, light!” Because “light” was the favorite suggestion during focus groups, we adjusted it based on the technical recommendation by adding “hey” in the beginning to enhance the recognition and prevent false activation of the system. We also modified the predefined lights into saving and loading positions, where users could save specific lighting conditions and load them later on. To activate the gesture system, in line with recommendations from Dietz et al [1], the user could hold the gesture they will use to edit the light settings for a certain period to perform an action. In this way, the probability of false positives will also be lowered, as it is less likely that surgical personnel will hold a specific gesture for a specific period. For instance, if the user wants to adjust the position of the light beam, they must hold the gesture for positioning the light first for a specific period. Once the system has been activated, the user is notified with auditory feedback that the gesture has been recognized, allowing the command (while maintaining the gesture) to be executed. We empirically set this time to 1.5 seconds after our initial testing sessions. The gesture system is deactivated when the command is executed until the next activation. Unlike Dietz et al [1], the user must only hold 1 gesture to activate and execute the command. In their approach, the activation gesture differed from the interaction gesture. To switch the lights on or off with hand gestures, users must hold an “L-shaped” hand gesture (Table 2) and move their hands in front, resembling the act of switching the lights on or off in the real world. Users must hold an “OK” gesture to position the light beam. The light would then follow the tip of their thumb and stop tracking once the hand is opened (Figure 3).

**Table 2:** Interactions in the virtual reality prototype for speech.

Interaction	Speech
System activation	“Hey, light!”
Light switch	“Lights on/off”
Light position	“20 centimeters left”
Light intensity	“Intensity 75”
Size of the illuminated field	“Angle 15”
Save or load positions	“Save/load position two”

This gesture was chosen as it would resemble grabbing the beam of light and moving it around. To modify light intensity, we implemented the “fist” gesture. Users have to move their fists up or down to increase or decrease the light intensity. We chose the “peace” gesture to save and load the positions. To save, the user has to move the hand forward and backward for loading. To choose a specific number to save or load, the hand has to be moved left to choose slot number 1 or right to choose slot number 2. For instance, if a user wants to save a specific position as position 2, they first have to move the hand forward and then to the right. This hand gesture was inspired by changing gears in a car. To change the size of the illuminated field, both hands have to be held as “L-shaped.” The closer the hands would get, the smaller the size, and vice versa. The complete list of interactions can be found in Table 2.

## Methods 3: VR User Study

### Overview

After adjusting the interaction concept, we implemented it in a VR prototype. In this prototype, one could adjust the SLS in a virtual OR using gesture and speech interaction. Both modalities were supported for every individual interaction. We designed the interactions based on the experts’ recommendations, technical considerations, and suggestions from the aforementioned literature. After developing the VR prototype, we consulted 2 surgeons and discussed the interactions that had been implemented with them before conducting the study. On the basis of their feedback, we made final adjustments to the prototype. We then tested the prototype with 2 novice users to identify and resolve further issues. For this study, we did not implement the functionality to return to the previous position to reduce study time and prioritize more essential interactions, as this was considered a minor feature.

### Implementation

**Figure 4:**
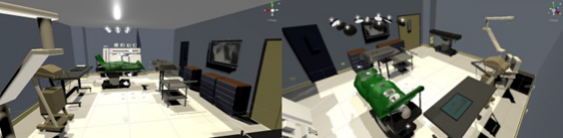
The virtual operating room used for the virtual reality study.

The prototype was implemented in Unity 3D [56]. We used Valve Index [57] VR glasses to conduct the study. A virtual OR was designed to simulate a natural environment (Figure 4). We used the Picovoice Speech-to-Text library [58] for speech recognition and the Valve Index’s 2 built-in cameras for gesture recognition using the hand-tracking SDK by HTC Vive [59]. A neural network classified the detected hands after matching features had been computed.

### Feedback System

Both auditory and visual feedback were developed for the prototype based on recommendations from previous research [1]. Brief auditory feedback was played after the system was activated and commands were executed. The audio clips were short and subtle to not annoy or overload users with auditory feedback.

**Figure 5:**
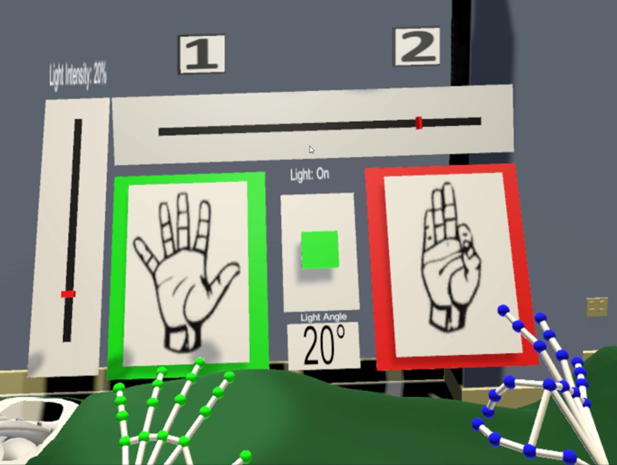
Visual feedback implemented in the virtual reality prototype.

The visual feedback system was aimed at providing supplementary information regarding the hand gestures so the participants could understand the interactions better. This feedback system would show participants their current recognized hand gestures for both left and right hands using 2 separate boxes (Figure 5). The border of the boxes representing hand gestures could be either red or green. The border would turn green when a gesture was held for 1.5 seconds. This meant that activation occurred, and the designated interaction could be done. The border is red if activation has not yet taken place. A green button in the center represents the light switch. The button would be pressed when users hold the designated gesture for the light switch (“L-shaped”) and move forward. Below this button, a number shows the size of the illuminated field. For this, we used degrees rather than the diameter or the radius of the light, as the diameter could change with repositioning on uneven surfaces. Furthermore, a slider on the left side showed the degree of light intensity. Another slider at the top showed the save and load slots.

### Participants

Six experts took part in our study. The information about the participants’ demographics can be found in Table 3. The participants included a chief physician, 2 senior physicians, and 3 assistant physicians. All were from the general and visceral surgery discipline. All surgeons regularly take part in operations (at least 2 times a week), besides one of the assistant surgeons, who takes part in operations once every 2 weeks. Only 2 surgeons had previous experience with VR, and 1 surgeon had experience with voice-based systems.

**Table 3:** Demographic information of the participants in the virtual reality study.

Participants	Sex	Age group (y)	Experience (y)
1	Male	56-60	26-30
2	Male	31-35	6-10
3	Male	31-35	1-5
4	Female	36-40	11-15
5	Female	36-40	6-10
6	Female	31-35	1-5

### Procedure

**Figure 6:**
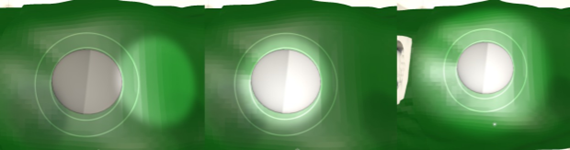
Examples of setting the illumination field and position of the light (left: wrong position; middle: correct setting; and right: field of illumination is too large).

**Figure 7:**
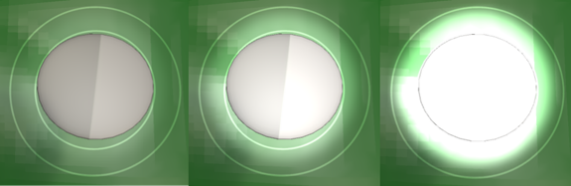
Examples of light intensity settings (left: too dark; middle: correct setting; and right: too bright).

In the beginning, the participants were informed about the procedure and risks of the study. After giving informed consent, the experts were given a detailed explanation of how the gesture and speech systems work and how the feedback system functions. They then spent 15 to 20 minutes familiarizing themselves with the VR environment. Furthermore, during this time, the participants were also asked to complete all the interactions at least once using both modalities to familiarize themselves with both speech and gesture systems. Subsequently, the experimenters explained the tasks for the study. Each participant had to perform the task in 3 rounds: once only using speech; once only using gesture; and in the last iteration, participants were free to choose the modality for each interaction. For the first 2 iterations, we changed the order of the modality for each participant to avoid order effects. In each iteration, the participants had to start by switching on the lights. They then had to position the light and adjust the illumination field, so it was positioned between the 2 drawn white rings (Figure 6). Then, they adjusted the light intensity by making it as bright as possible while not so bright that the gray tones could no longer be distinguished (Figure 7). Finally, they had to save the light settings and turn off the lights. After each iteration, participants answered a series of interview questions. The questions addressed aspects such as learnability, memorability, familiarization, audio and visual feedback systems, suggestions for improvement, and opinions on individual commands. Each session took approximately 40 to 50 minutes.

## Results 3

### Overview

Our results showed that participants found the concept compelling and helpful. All experts stated that they could imagine using the system regularly in the OR as a new system or as an add-on to the conventional lights. We witnessed that the experts often prioritized one modality over the other. Three participants suggested that gestures were generally more intuitive and easier to use, while others believed that speech was more convenient and faster to learn. One reason mentioned for this prioritization was that it was easier to master 1 than the 2 modalities. Two participants pointed out that the touch-free aspect of the interactions was a great advantage and very beneficial in the OR. They mentioned that such a system helps keep sterility and avoids asking other staff members to perform specific actions. On the other hand, 2 experts believed that the system is still not ready to fully replace a conventional SLS due to error rates and the time it takes to execute the commands, as it would be a potential risk in time-critical or emergency situations such as stopping heavy bleeding. They pointed out that the precision needs to be extremely high and the interactions even quicker.

### Feedback Systems

All participants agreed that the audio feedback was helpful. Four experts mentioned that such feedback is necessary, especially for triggering the commands. Two participants noted that the auditory feedback could be turned off after a training phase for visible interactions such as positioning the light. However, 2 others mentioned that they could not imagine the system working well without auditory feedback. Participants found the activation tone of the speech system more helpful than the confirmation tone because the successful execution of the command is mainly confirmed by the visible changes in the light (with the exception of the save and load command). Regarding the visual feedback interface, 5 participants found it to be helpful, especially in the beginning. Four experts mentioned that they could use the system without visual feedback after a short training. However, 1 participant found the visual feedback disruptive and suggested only using auditory feedback.

### Interactions

All but one participant found the gesture system intuitive and easy to learn. All agreed that with a short familiarization phase, one could understand and learn to interact with the system. However, 2 participants indicated that our familiarization phase was too short for the gesture interactions, and they recommended prolonging that to 30 to 60 minutes. Two participants found the execution of gestures to be ergonomic and quick. Three participants highlighted that knowing the commands from similar activities helped them to learn the interactions faster and the execution of the commands easier. Five experts believed that gesture interaction was more appropriate for positioning the light, as it was more precise. One participant found the load and save gesture command rather tricky and unintuitive. Another participant noted that it was unclear how to stop the hand tracking after performing the command. One participant found the gesture recognition not precise enough. The error rate for this user was higher than that of all other participants. Similar to the gesture system, all participants perceived speech commands as intuitive, fitting, and easy to remember. Two participants added that they particularly liked that the commands were short, which can be advantageous when used in the OR. One participant highlighted the high accuracy of the speech system. Two participants specifically praised the wake word “Hey, light!” and found it appropriate and fitting. One participant remarked that the trigger functions or functionality switches [11,38] are very suitable with voice commands because performing them requires little cognitive and physical effort (eg, light switch). A major criticism that was pointed out by 3 participants was about setting continuous parameters with speech, such as positioning the light beam. They found such interactions overly complex and confusing. One expert noted that estimating the numbers as measurements needed for the voice command is difficult. All participants wore medical masks during the study. We did not observe any negative impacts caused by wearing the mask. One of the participants believed that using the German language would be more convenient for the speech system. Others found English to be appropriate and sufficient. One participant experienced difficulties with the speech commands due to their accent.

### Modality Preferences

Participants’ reasons for choosing a modality over another were based on the number of commands needed for execution, the speed of the interaction, the cognitive and physical effort, and the precision of the commands. All but one participant used a mixture of both speech and gestures to go through the tasks in the last iteration. Table 4 shows the chosen modality of all participants for individual interactions in the third iteration. Among the 5 subtasks were 2 trigger functions and 3 continuous parameter settings. We observed that parameters were preferred to be set by gesture, as the dynamic feedback would help to interact more precisely. For such interactions, multiple speech commands were often needed to set the parameters accordingly. In the subsequent interview, we asked the participants why they chose a particular modality for each command in the last iteration. During these interviews, we found that, although participants might have preferred one modality over the other, 2 participants chose the less favored one to give it another chance. One participant who experienced problems using the speech system due to unclear English pronunciation performed all the tasks in the last iteration with the gesture. One participant indicated that they mainly worked with speech, as the familiarization phase was too short for them, and they forgot how to carry out the command with the other modality. All participants stated they could use the system entirely without further help after a short training period. Two participants stated that having both modalities simultaneously during surgeries is beneficial. One said that they would be able to use voice commands when their hands are busy or gesture when the noise level in the room is very loud. No participant expressed a negative characteristic of using both modalities simultaneously. Participants particularly emphasized the advantage of dynamic feedback and fine motor skills when setting continuous parameters with gestures, as well as speed and the low cognitive and physical effort in trigger functions when using speech.

**Table 4:** The selected modalities for each interaction in the third iteration of the virtual reality study.

Interaction type	Speech	Gesture
Light switch	5	1
Position	1	5
Intensity	3	3
Field of illumination	0	6
Save or load	4	2

## Methods 4: Living Laboratory Study

### Overview

The interaction concept was implemented in a prototype to be tested in a living laboratory. Similar to the VR study, participants could adjust the SLS in a living laboratory using gestures and speech, where every interaction could be made with both modalities. In this study, we used smart lighting modules introduced by Mühlenbrock et al [20] to test our interaction concept. This novel lighting system is specifically designed for the OR, which uses an array of small lighting modules mounted on the ceiling [20].

### Smart Ceiling Lights

**Figure 8:**
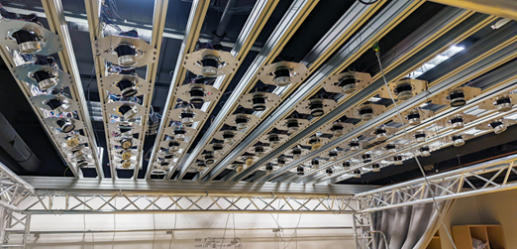
Smart ceiling lights used for the living laboratory study, consisting of an array of small lighting modules.

Each module is equipped with 2 motors for directional adjustment. The modules are arranged in a 7×8 grid with distances of 0.36×0.35 m and are controlled by a central computer that adjusts their intensity and orientation (Figure 8). To minimize shadows, the system uses geometry recognition of objects and personnel within the surgical area via 3 Kinect (version 2) [60] sensors, which are registered with each other and with the room. However, the automatic shadow prevention feature was disabled during the study. The lighting system provides an application programming interface that accepts commands for the absolute target position of the light in 3D space, relative adjustments to this position, and changes in lighting intensity. It then adjusts the motors and intensity of the lights accordingly. While individual modules are capable of providing up to 50 klx, compliance with medical standards limits the system to a maximum of 160 klx. The paper by Mühlenbrock et al [20] provides further details on the ceiling lights.

### Interaction Concept Integration

We integrated our interaction software with the lighting system application programming interface to be able to interact with the lights using our touch-free concept. The prototype was implemented in Python (Python Software Foundation). We used Kinect Azure [61] for tracking hand gestures and used the Picovoice Speech-to-Text library [58] for speech recognition. A neural network classified the detected hands after matching features had been computed.

Due to the limitations regarding the motors in individual light modules, we were not able to change the field of illumination adequately. Therefore, we excluded this interaction from this study. The rest of the interactions were identical to the ones in the VR study (Table 2).

Moreover, in a realistic surgical environment, multiple hands may appear within the camera’s field of view, complicating accurate hand recognition. To address this, we used the Kinect RGB camera and trained the system to track hands wearing a specific color of gloves—in this case, blue. This way, we could ensure that the system only tracks the designated surgeon’s hands to adjust lights. Testing confirmed that only hands with blue gloves were detected. A notable advantage of this method is its ability to restrict access to authorized users based on glove color. However, a limitation arises when gloves become bloodstained, as red-tinted areas may hinder recognition. To mitigate this issue, the algorithm could be adapted to reassign red pixels to a skin-like color space to ensure consistent recognition.

### Participants

For this study, we recruited 12 experts. Other than 1 medical student, all other participants were surgeons or assistant surgeons, and they regularly took part in operations (at least 2 times a week). The information about the demographics of the participants can be found in Table 5. All participants were from the general and visceral surgery disciplines.

**Table 5:** Demographic information of the participants in the living laboratory study.

Participants	Sex	Age group (y)	Experience (y)
P1	Female	21-25	1-5
P2	Male	41-45	16-20
P3	Male	46-50	21-25
P4	Female	26-30	1-5
P5	Male	31-35	6-10
P6	Male	26-30	1-5
P7	Male	31-35	1-5
P8	Male	26-30	1-5
P9	Female	41-45	11-15
P10	Male	36-40	11-15
P11	Male	31-35	6-10
P12	Female	26-30	1-5

### Procedure

**Figure 9:**
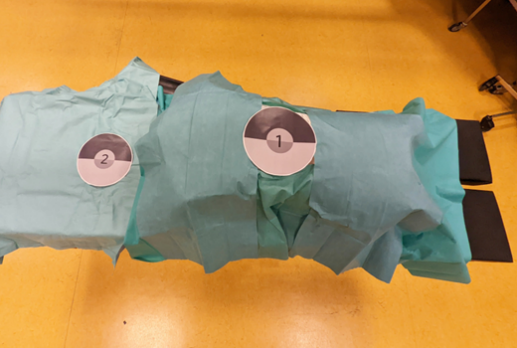
Setup of the user study in the living laboratory.

After a short welcoming conversation, participants were briefed about the study procedure and gave informed consent. All participants were given a detailed explanation of how the interaction with the system works and how to use both modalities of gesture and speech. Participants then spent around 10 minutes familiarizing themselves with the system by performing all the supported interactions at least once using both modalities. Afterward, the experimenter explained the tasks to perform for the study. Similar to our VR experiment, each participant had to perform the task in 3 rounds. However, this time they could choose which modality to use for individual interactions in every iteration. For each iteration, we used 2 circular position marks, numbered mark 1 and mark 2 (Figure 9). Participants had to position the light on each ring, save that position with the mark’s associated number, and switch between the 2 positions by loading the previously defined positions. The light intensity had to be set differently for each mark. One had to have a higher intensity and one, a lower intensity. Adjusting the size of the illuminated field was not considered in this study due to technical limitations with the physical lights in the living laboratory. The participants chose the order of the tasks to perform, and the experimenters suggested no strict order. At the end of each iteration, participants took a short break and continued with the next one. Once the last iteration was through, a semistructured interview was conducted where experts answered a series of questions about the overall system, learnability, memorability, familiarization, audio feedback, suggestions for improvement, and opinions on individual commands. Sessions took approximately 30 (mean 31.91, SD 4.85) minutes.

## Results 4

### Interaction Ratings

**Figure 10:**
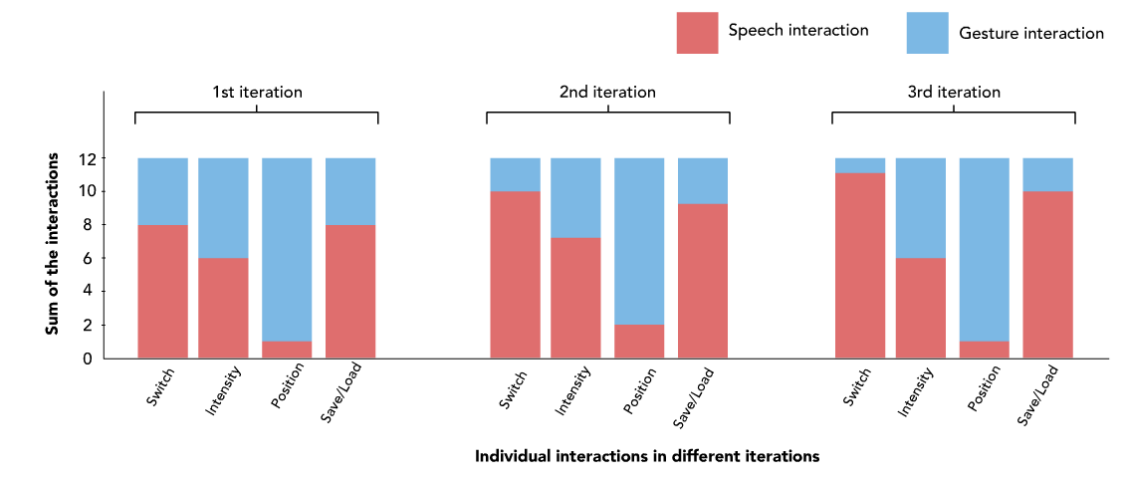
Bar chart showing the experts’ selected modality for each interaction on all 3 iterations.

Our results indicated that most participants (11/12, 92%) found both speech and gesture interactions appropriate for their intended actions. Most participants (9/12, 75%) envisioned using such a system in the OR regularly. All participants agreed that, with a brief training period, they could use the system without needing ongoing technical support. While 2 participants suggested that the training might take up to a week, the remaining participants believed it could be completed in under an hour. They described the interactions as “fast and easy” (P3). We witnessed that the experts’ performance improved over time. We saw that they worked easier with the system in the second and third iterations compared to their initial attempts. This trend suggests a positive learning effect with increased exposure to the system. Initially, during the first 2 iterations, participants were open to using both speech and gesture modalities for various interactions. However, by the final iteration, clear preferences for specific modalities had emerged depending on the task (Figure 10). For certain interactions, distinct modality preferences became apparent. Participants found speech more suitable for trigger functions, such as switching lights or saving and loading lighting conditions. Conversely, gestures were preferred for continuous parameters, such as positioning the light. Opinions on controlling light intensity were divided: half of the participants found gesture interactions more intuitive, while the other half preferred using speech. All participants considered audio feedback as highly crucial for their interactions. A few participants (3/12, 25%) suggested using distinct sounds for different actions to help differentiate them more clearly. Participants found audio feedback particularly beneficial for specific tasks, such as positioning, as it provided essential information about the system’s current state and alerted them when an action was not recognized.

Despite general satisfaction with individual interactions, participants provided several recommendations for adjustments and suggested new interaction features. Two participants recommended using shorter speech commands for adjusting light intensity. They proposed a simplified system where intensity is set on a scale of 1 to 10 rather than using percentage values. One participant (P5) suggested adding a feature to disable gesture tracking via speech commands to prevent misrecognition and unintended hand tracking. Participants also recommended the ability to revert to a previous light configuration. There were suggestions to combine modalities for specific interactions. For instance, 1 expert proposed a mixed approach for the save and load function: initiating the action with a gesture and then using speech to assign it to a specific slot (eg, “Save two”). However, 4 participants found the gesture-based save or load interaction unintuitive and overly complex. Two participants noted that this interaction was cognitively demanding because it required memorizing which position corresponded to each number. One participant suggested using specific names for save slots (eg, “save stomach”) instead of numerical labels. In addition, 5 participants explicitly mentioned that using speech commands for positioning was inappropriate. They found it required excessive effort and approximation, making it less effective compared to the gesture.

### Experimenter Observations

We observed that the field of view of the depth camera used to track hand movements positioned above the surgical bed was limited. Experts noted the desire to move the surgical light into zones that fell outside this camera’s range. This limitation could be addressed by integrating multiple cameras, which would expand the tracking area and potentially enhance gesture recognition accuracy. Despite the system’s capabilities, we still encountered several recognition errors for both speech and gesture modalities. To ensure reliability, these systems require better training and increased accuracy, especially in a high-stakes environment like an OR where patient safety is critical. The current system’s precision needs to be enhanced to meet the strict demands of surgical settings. We found that participants’ proficiency with the system improved over time with increased use. This suggests that future implementations should include longer and more comprehensive training sessions to help users become adept at operating the system efficiently. Four participants reported that the gesture-based positioning of the surgical light was slow. This issue stemmed from the limitations of the motors used in the light modules, which caused a noticeable delay in moving the light. Addressing these mechanical limitations could improve the system’s responsiveness and enhance the user experience.

## Discussion

### Principal Findings

In this work, we examined a hands-free solution for controlling an SLS in the OR. In an iterative design process, we developed a system that uses speech and gestures as interaction modalities to control the SLS. We observed that surgeons and practitioners found such a system highly valuable and practical. They believed that this system could certainly support sterility and give them more direct control over the SLS.

Our results highlighted that the combination of modalities was a quite helpful approach, supporting the previous literature [10,29,42]. We witnessed certain interactions (specifically, triggers) that were much better suited to be performed by speech and others better suited with gesture (continuous parameters), in line with previous work [11,38]. In line with the recommendation from Mentis et al [11], our participants also suggested having the opportunity to be able to use both modalities for each interaction to have higher flexibility. We witnessed that experts often preferred one modality over the other, largely based on perceived cognitive load and familiarity with the modality. Participants’ opinions differed on which modality they found less cognitively demanding; some considered speech interaction easier due to its directness and hands-free nature, while others preferred gestures for visual immediacy. These findings highlight the potential of multimodal systems to enhance interaction effectiveness. The clear observation that certain tasks are better suited to one modality over another highlights the advantages of leveraging the strengths of both speech- and gesture-based interactions. Although mastering both interaction methods might initially pose a cognitive challenge for users, we believe this can be mitigated through brief technical training and the design of intuitive interactions and significantly reduce cognitive demands over time. Moreover, multimodal systems also serve as a critical redundancy layer—ensuring functionality even if one modality fails. In addition, providing users with the flexibility to choose their preferred modality fosters greater satisfaction and increases the likelihood of broader adoption.

The list of functions we planned for the adjustment of the lights was consistently found to be practical and useful by participants. Notably, participants highly favored predefined light settings, aligning with findings from previous research [3], as these settings simplify operations and save time for the personnel. The individual interactions were mainly found to be intuitive and usable. However, certain interactions that were not matched well with their modality were criticized, for example, saving and loading light settings with gesture. As this is considered a trigger function, speech seems to be the more intuitive modality to use. Participants found the freehand control aspect of using speech quite helpful. This meant the surgeons could use both hands at all times and focus on their primary tasks, as pointed out by previous work [38,39]. Furthermore, it is common that during surgeries, multiple hands are actively working on the patient, making it challenging to identify the correct hand for gesture control. Another concern with gestures is that they can cause collisions, a problem that does not occur with speech. On the other hand, speech is less appropriate for fine adjustments and more exact changes, as seen in our evaluations. In addition, a loud OR environment and problems with speech recognition, such as the accuracy for nonnative speakers or people with accents or certain dialects, could also be raised. These issues further emphasize the benefits of a multimodal system, where challenges with one modality can be mitigated by leveraging the strengths of the other.

A key advantage of our proposed system was the enhanced agency it could offer to the main surgeon. This was particularly appreciated when the surgeons’ hands were occupied, as they could still control the lights, providing them with higher control and efficiency. Experts highlighted the benefit of eliminating the need to rely on additional personnel for adjustments, potentially reducing errors and saving time [2].

Throughout our examination, we observed that the biggest concern raised by the experts was the technological limitations, such as accuracy and functionality problems with the systems. In different stages of our evaluation, participants faced certain challenges with inaccuracies where the developed system did not behave as the experts intended it to do. The experts repeatedly highlighted that in such a sensitive context where patients’ safety could be impacted, there is no room for error. Hence, the system ideally has to function error-free with perfect efficacy. As previous work highlighted, events that require unnecessary actions from the surgeons could hinder their performance [2]. It comes as no surprise that systems deployed in sensitive environments, such as health care, must prioritize reliability and error mitigation. Experts’ emphasis on error-free operation highlights the unique stakes in surgical contexts, where minor inaccuracies could risk patient safety. In such high-stakes applications, designers must aim for the highest technical accuracy and consider appropriate backup methods and error recovery means to safeguard against unanticipated failures. Several technical concerns were highlighted, including the high noise levels in the OR and worries about their potential impact on speech command accuracy, as well as the challenges of achieving the high precision required for fine adjustments. Addressing these challenges is essential, and future research should focus on developing solutions that specifically mitigate these issues to ensure reliable system performance. Moreover, we observed that few participants did not trust the technology to work seamlessly due to old experiences or unfamiliarity with the technology (eg, “speech will not work well!”). Our participants highlighted that the lack of trust in the technology could raise the stress levels of the surgeons. Skepticism stemming from users’ past experiences or unfamiliarity with the technology underscores the importance of fostering trust through transparency, education, and hands-on training with novel systems. Bridging the gap between perceived and actual system reliability through effective communication and usability testing is essential for promoting user trust and ensuring successful integration. A gradual adoption in low-risk contexts (such as VR simulation) can build confidence before full integration in critical tasks.

### Limitations and Future Work

Our results offer valuable insights into using a touch-free interaction approach for controlling the SLS in the OR. Nevertheless, our work has certain limitations that require acknowledgment.

In our iterative evaluation, we mainly relied on qualitative approaches to assess our concept and interaction system. This provided us with in-depth insights into user perceptions, behaviors, and subjective experiences, enabling us to refine and improve the system based on detailed feedback and observations. However, for future work, incorporating quantitative approaches will be crucial to measuring usability, cognitive load, and user experience more objectively. By using metrics such as task completion times, error rates, physiological measurements, and standardized questionnaires, we can obtain a comprehensive understanding of the system’s performance and its impact on users, ensuring a more robust and reliable evaluation. Moreover, our designed system was still a prototype, and participants encountered technical challenges at various stages. Future work should also focus on better training the systems to improve recognition rates, providing a more reliable foundation for such interaction methods.

Our studies primarily consisted of laboratory experiments and interviews. As an initial approach, this selection of methods is reasonable, providing controlled conditions to refine the system and gather preliminary insights. Nevertheless, testing the system in more complex environments and realistic situations—such as in noisy environments, with multiple hand-in scenarios, and across various surgical disciplines—would be beneficial. This would help us observe and address potential challenges and limitations, ensuring the system’s robustness, adaptability, and effectiveness in real-world applications.

Finally, in our experiments, we observed that longer training sessions with the system could have led to better performance from the experts. Therefore, we recommend extended familiarization phases to allow users to become more acquainted with the system, enhancing their proficiency and effectiveness. Future work could also explore the optimal duration and structure of these training sessions.

### Conclusions

This work sought to develop an intelligent interaction concept for controlling an SLS. We designed a touch-free interaction concept using speech and hand gestures to control the SLS. In an iterative process, using a user-centered design approach, we used a 5-step design process including a literature review and OR visits, interviews with surgeons and assistant surgeons, focus groups with the OR personnel, a VR evaluation, and a living laboratory study. Our findings suggest that experts find our interaction concept highly useful and beneficial for the OR. Despite facing technical challenges, surgeons saw great potential for such a touch-free approach and could imagine using it regularly in the OR. Moving forward, further refinement and validation of such a multimodal system will be essential to fully realize its potential in real-world surgical settings.

## Appendix

Multimedia Appendix 1 Supplementary figures and illustrations. This appendix includes all figures referenced throughout the paper.

## Abbreviations

NUI: natural user interface
OR: operating room
SLS: surgical lighting system
VR: virtual reality
