# Supplementary figures and images for: Using Gesture and Speech to Control Surgical Lighting Systems: Mixed Methods Study
Source: JMIR Hum Factors. 2025 May 12;12:e70628. doi: 10.2196/70628 (PMC12107204; doi:10.2196/70628)

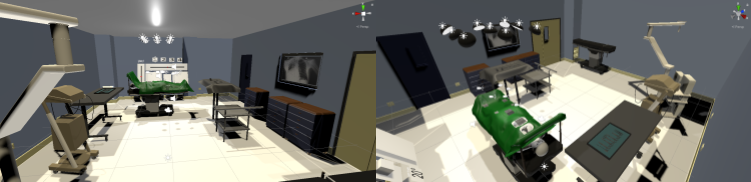

Supplement: Multimedia Appendix 1 [file humanfactors_v12i1e70628_app1.zip › Figures/VROPRoom.png]

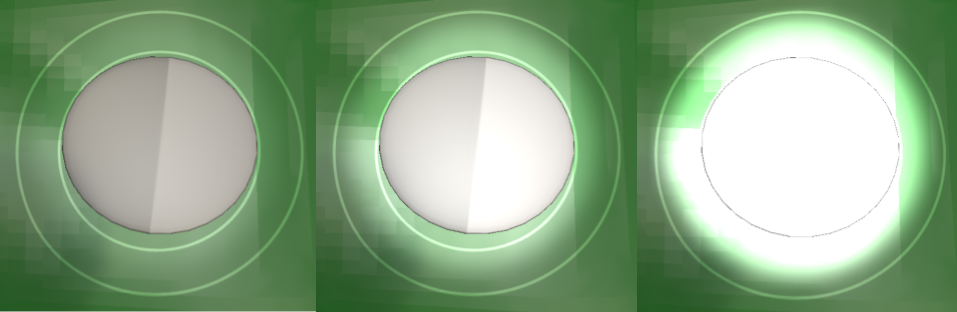

Supplement: Multimedia Appendix 1 [file humanfactors_v12i1e70628_app1.zip › Figures/Intensity.png]

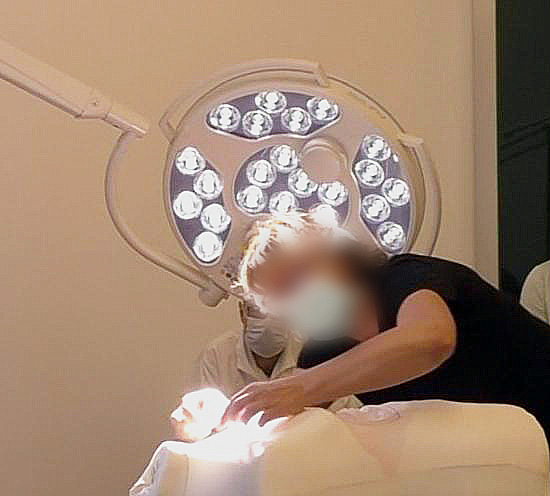

Supplement: Multimedia Appendix 1 [file humanfactors_v12i1e70628_app1.zip › Figures/2ndFocusGroup2.jpg]

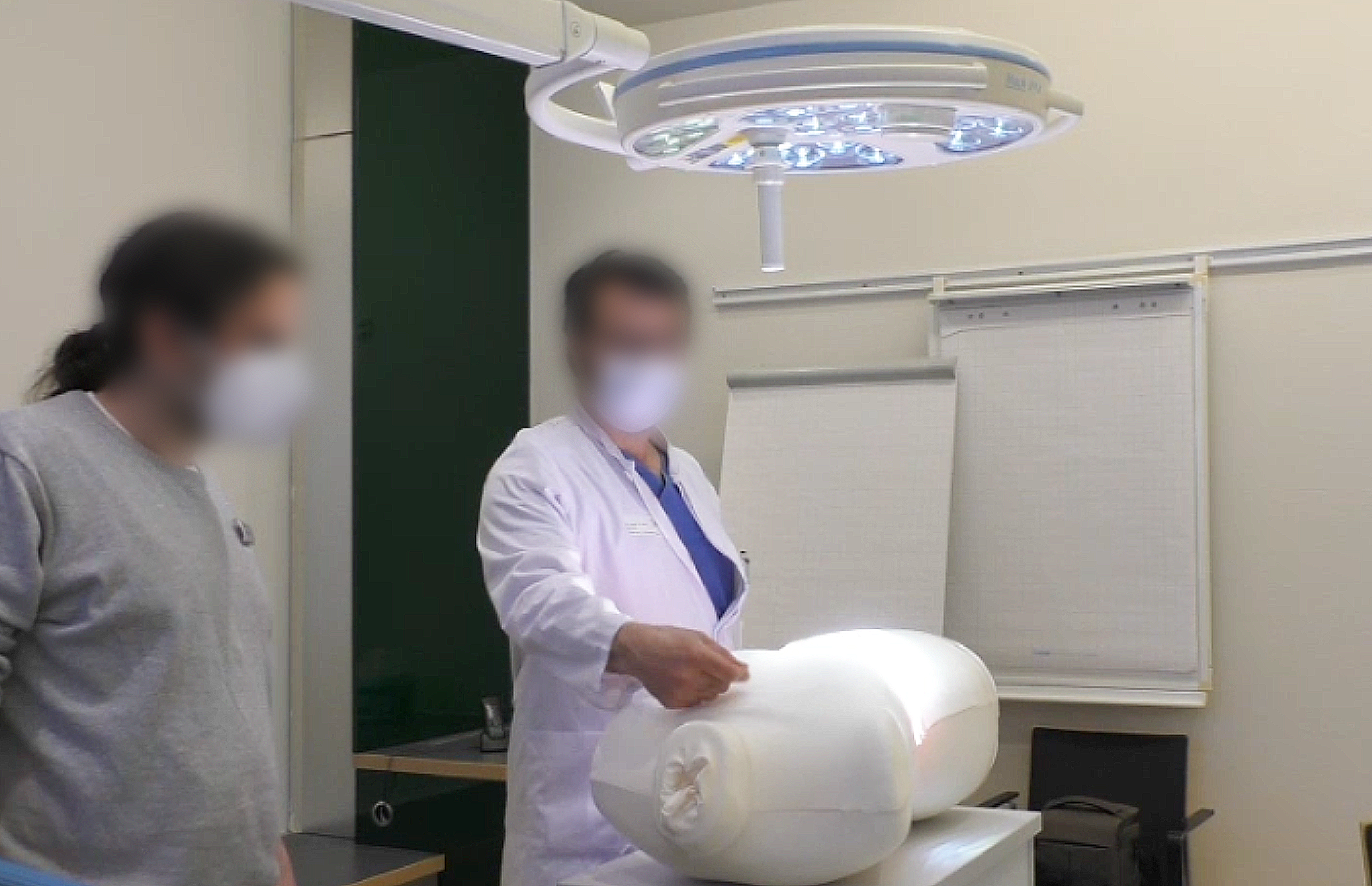

Supplement: Multimedia Appendix 1 [file humanfactors_v12i1e70628_app1.zip › Figures/1stFocusGroup.png]

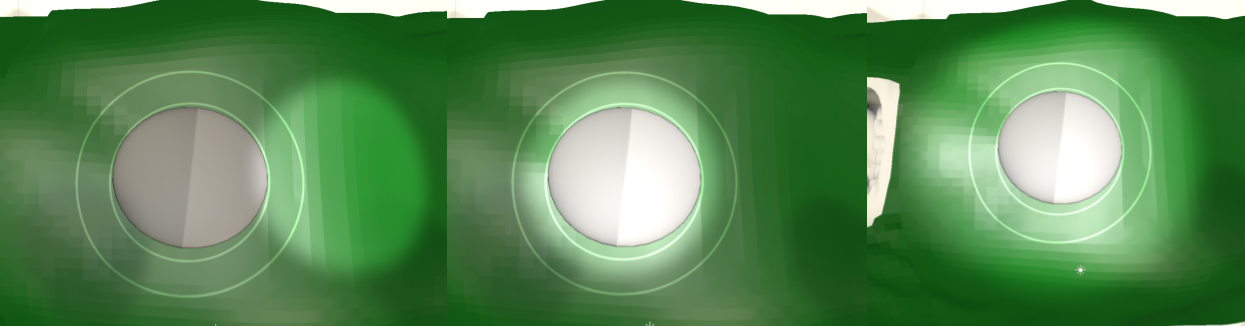

Supplement: Multimedia Appendix 1 [file humanfactors_v12i1e70628_app1.zip › Figures/Position.png]

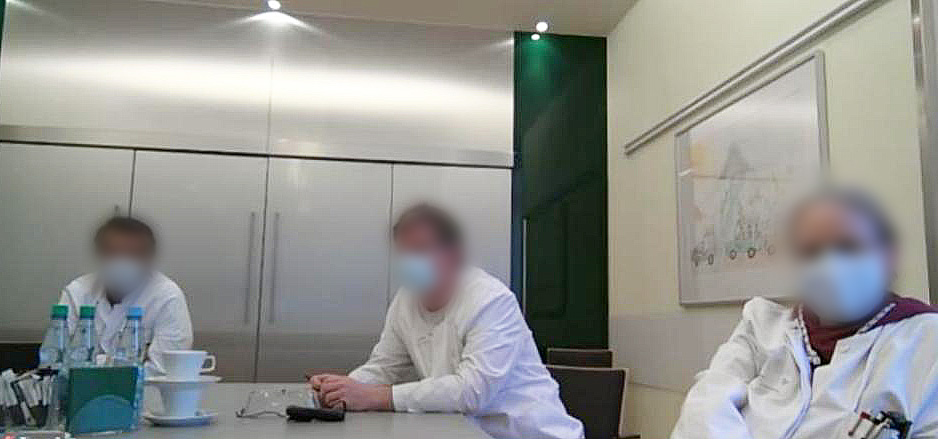

Supplement: Multimedia Appendix 1 [file humanfactors_v12i1e70628_app1.zip › Figures/1stFocusGroup.jpg]

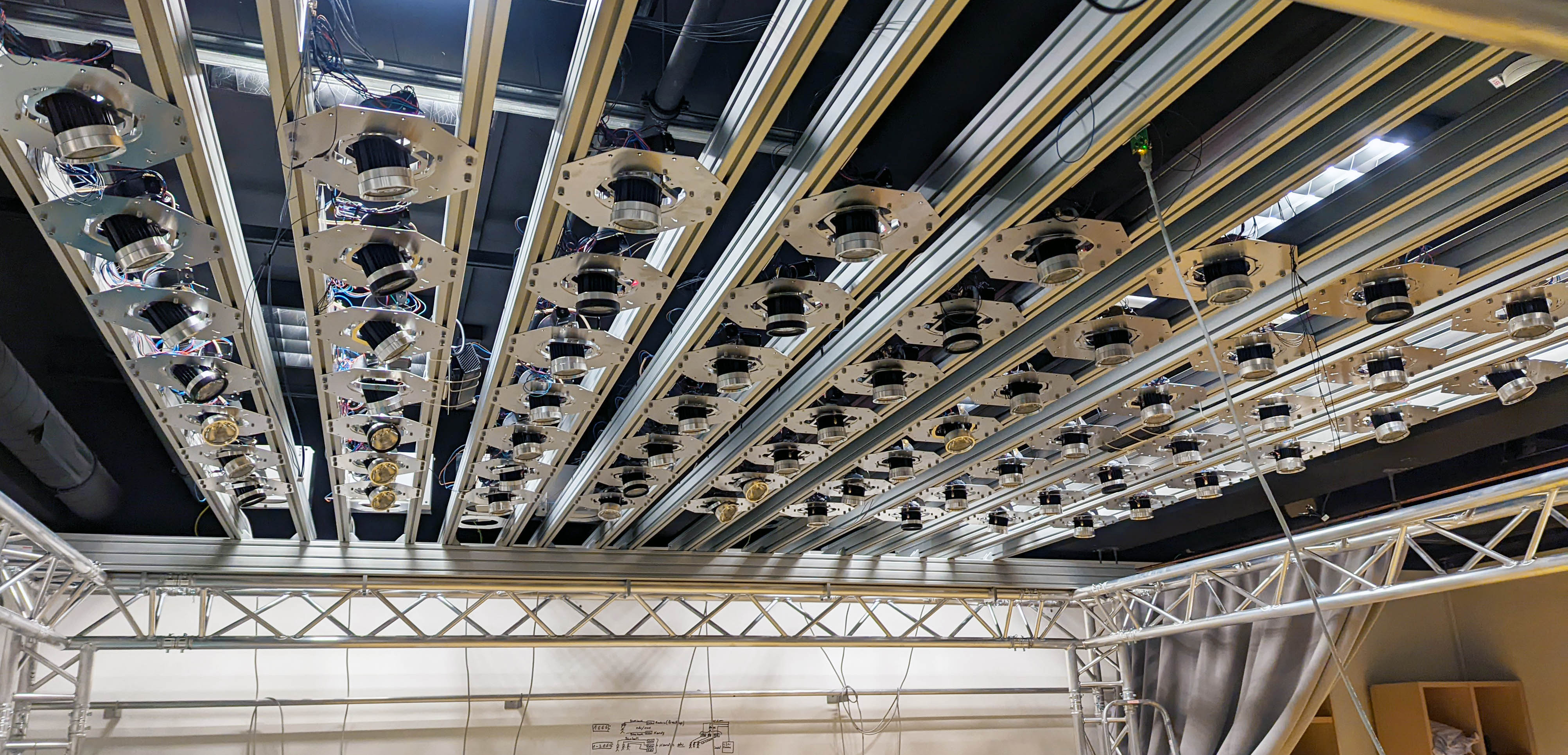

Supplement: Multimedia Appendix 1 [file humanfactors_v12i1e70628_app1.zip › Figures/ceilingLights.jpg]

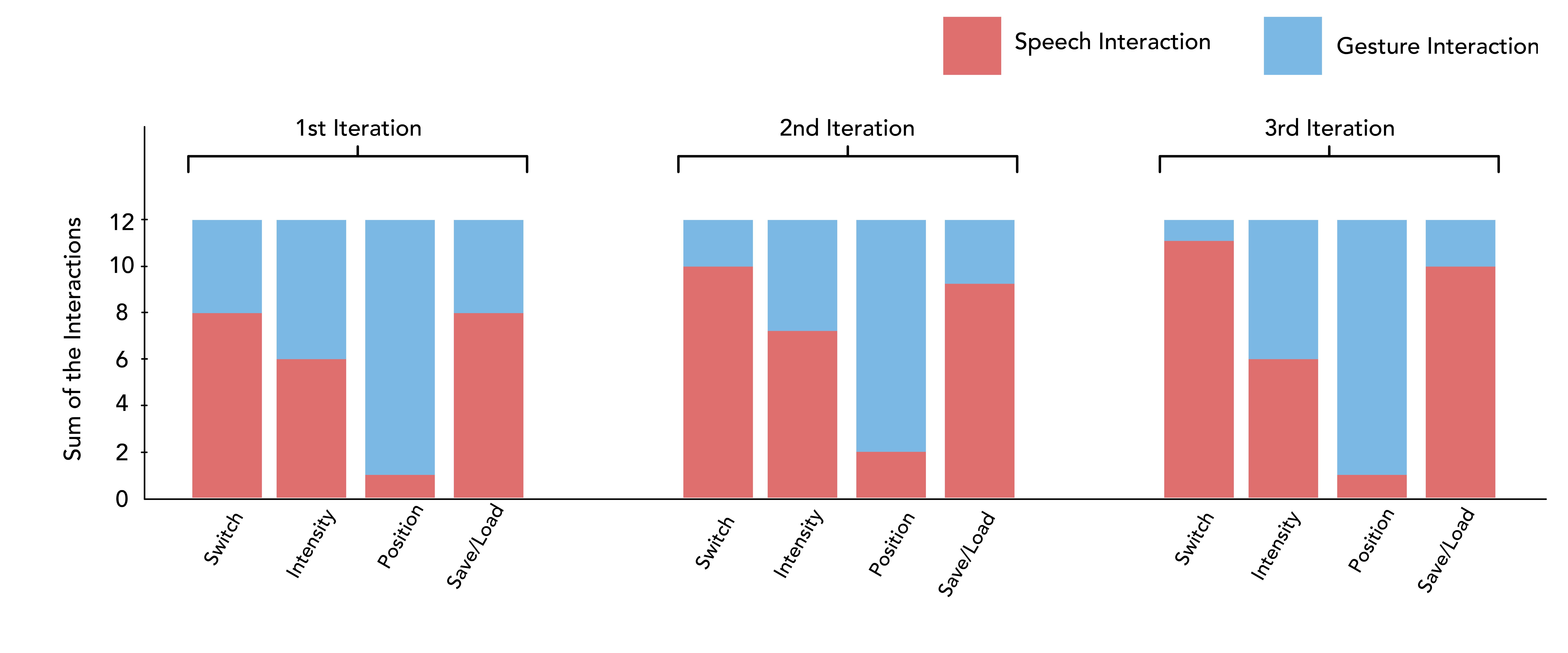

Supplement: Multimedia Appendix 1 [file humanfactors_v12i1e70628_app1.zip › Figures/Interactions_BarChart.jpg]

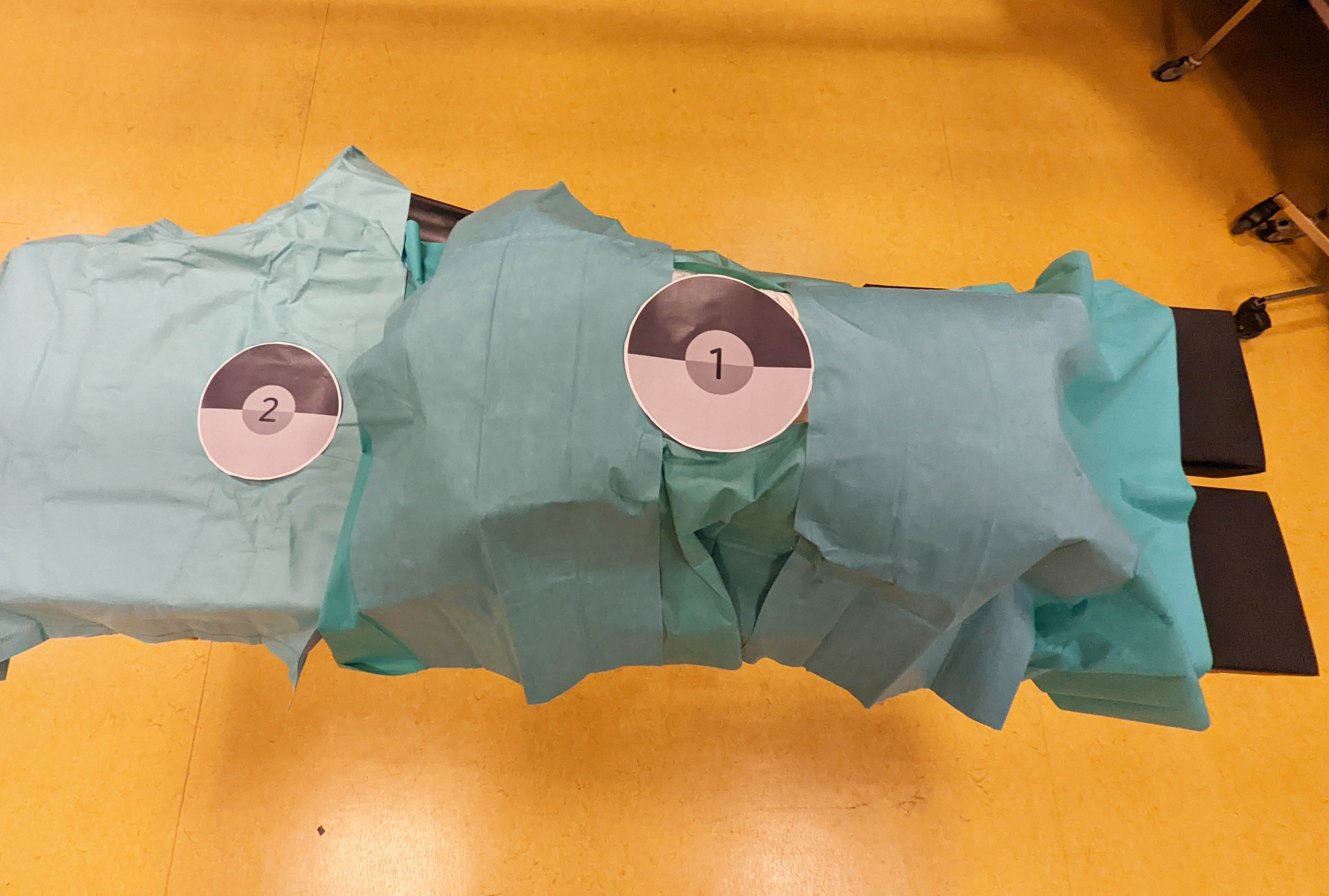

Supplement: Multimedia Appendix 1 [file humanfactors_v12i1e70628_app1.zip › Figures/MainStudySetup2.jpg]

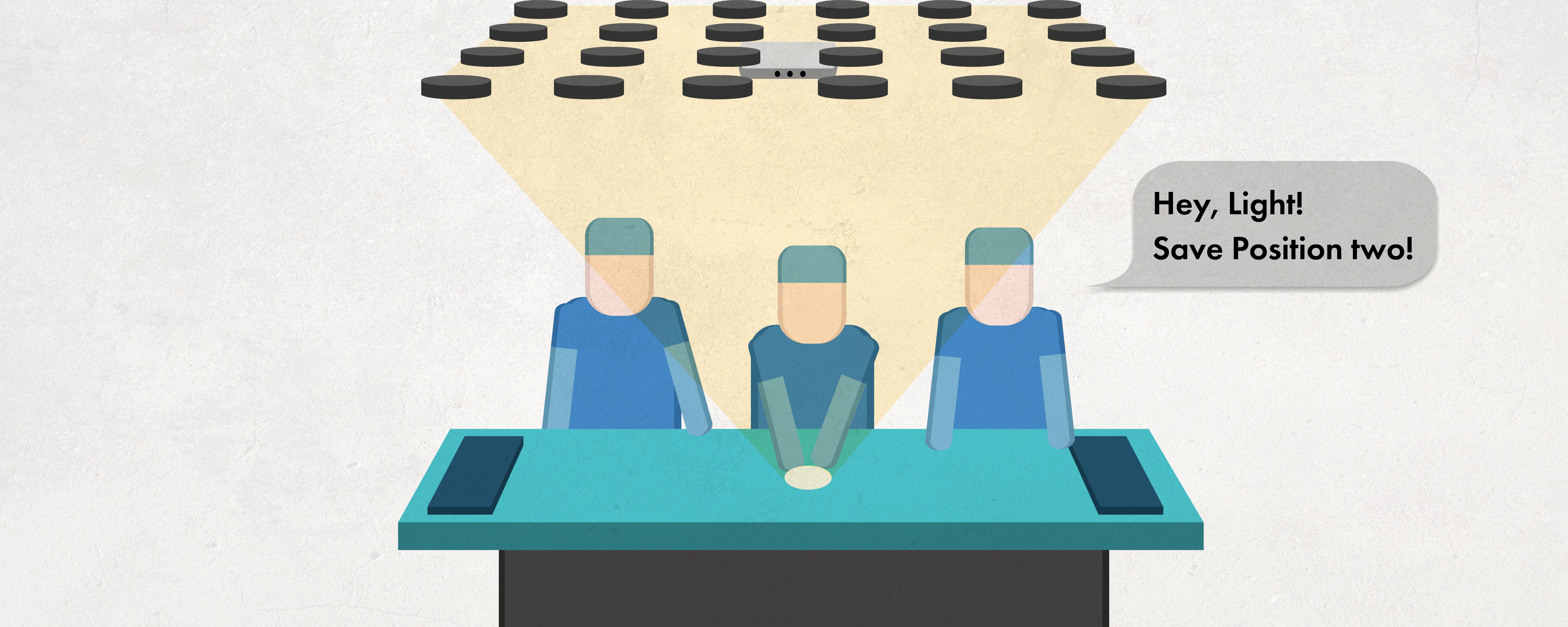

Supplement: Multimedia Appendix 1 [file humanfactors_v12i1e70628_app1.zip › Figures/SmartOT_Sketch.jpg]

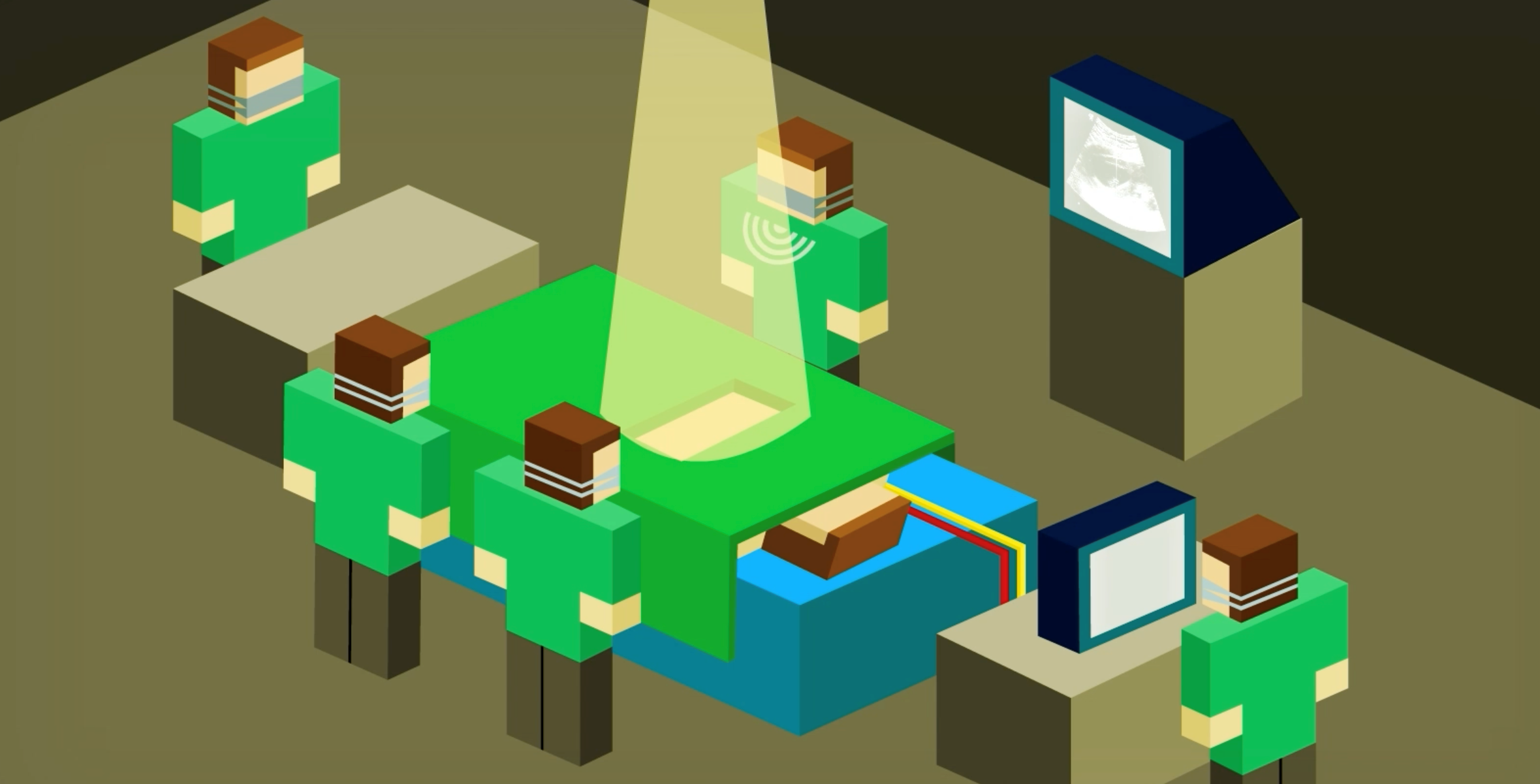

Supplement: Multimedia Appendix 1 [file humanfactors_v12i1e70628_app1.zip › Figures/ConceptAnimation3.png]

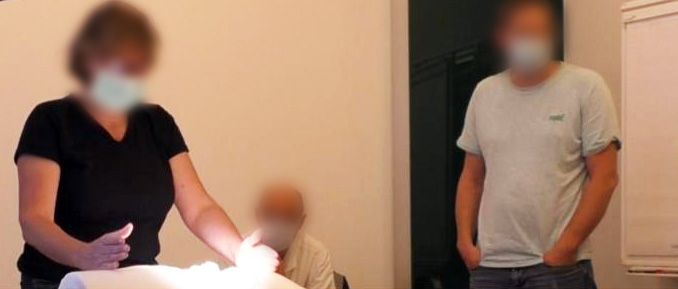

Supplement: Multimedia Appendix 1 [file humanfactors_v12i1e70628_app1.zip › Figures/2ndFocusGroup.jpg]

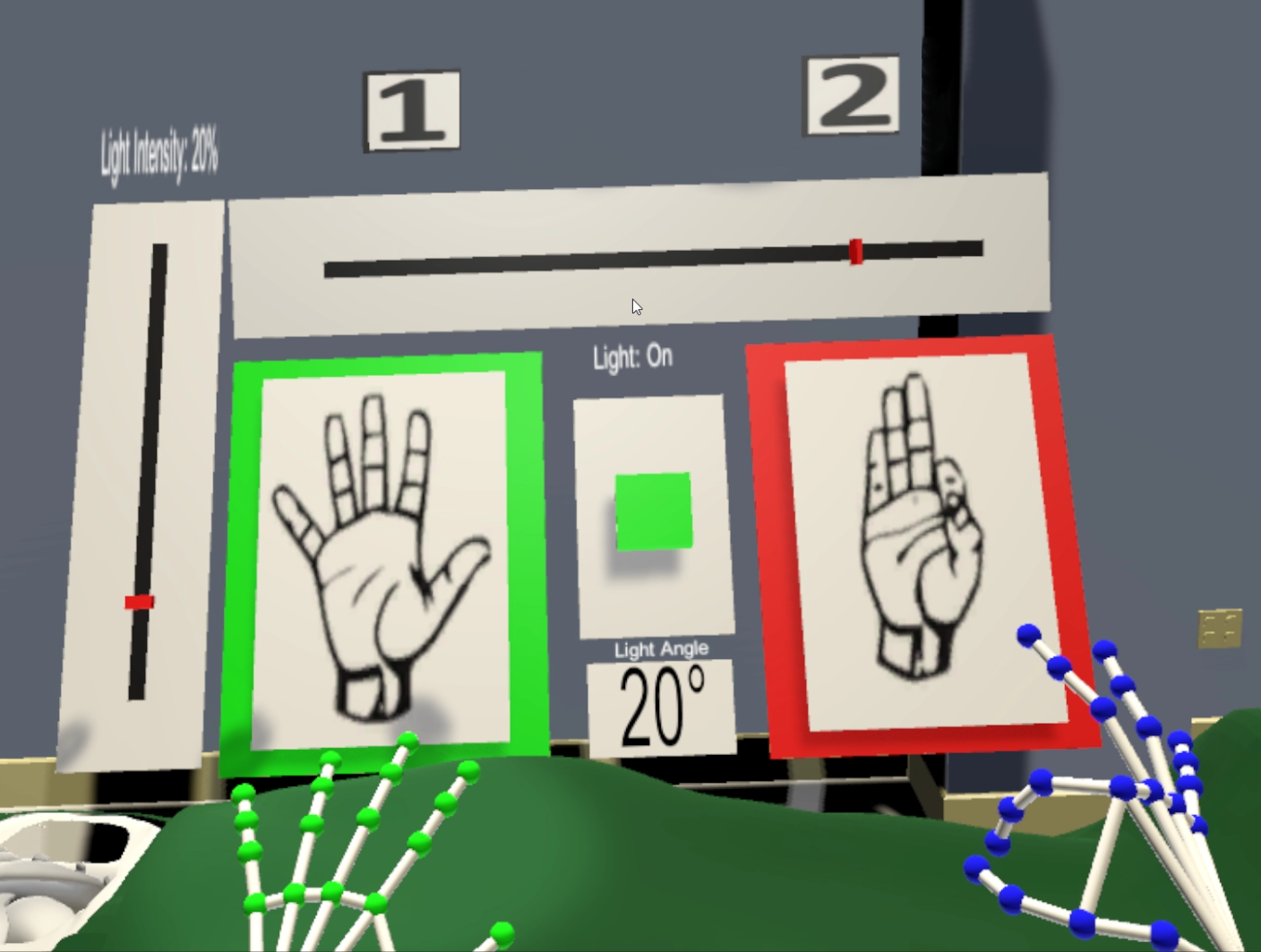

Supplement: Multimedia Appendix 1 [file humanfactors_v12i1e70628_app1.zip › Figures/visuellesFeedbacksystem2.png]

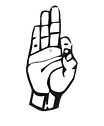

Supplement: Multimedia Appendix 1 [file humanfactors_v12i1e70628_app1.zip › Figures/Hands/Ok.png]

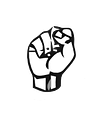

Supplement: Multimedia Appendix 1 [file humanfactors_v12i1e70628_app1.zip › Figures/Hands/Fist.png]

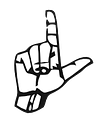

Supplement: Multimedia Appendix 1 [file humanfactors_v12i1e70628_app1.zip › Figures/Hands/L.png]

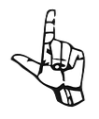

Supplement: Multimedia Appendix 1 [file humanfactors_v12i1e70628_app1.zip › Figures/Hands/LLeft.png]

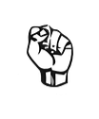

Supplement: Multimedia Appendix 1 [file humanfactors_v12i1e70628_app1.zip › Figures/Hands/FistLeft.png]

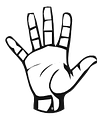

Supplement: Multimedia Appendix 1 [file humanfactors_v12i1e70628_app1.zip › Figures/Hands/OpenHand.png]

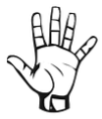

Supplement: Multimedia Appendix 1 [file humanfactors_v12i1e70628_app1.zip › Figures/Hands/OpenHandLeft.png]

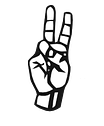

Supplement: Multimedia Appendix 1 [file humanfactors_v12i1e70628_app1.zip › Figures/Hands/Peace.png]
